# Efficacy and Safety Comparison of S-1 and Capecitabine in Metastatic Colorectal Carcinoma: A Systematic Review and Meta-Analysis

**DOI:** 10.7759/cureus.90806

**Published:** 2025-08-23

**Authors:** Abdullah I Alhamzani, Rayan H Almutairi, Osama Y Almajhadi, Duaa A Aljuhaymi, Sawsan A Alghamdi, Layan K Alsubhi, Elaf M Emam, Shuruq A Albouq, Razan H AlQahtani, Jawad Y Bukhari, Faris T Alomari, Sarah H Alzaid, Rolina Al-Wassia

**Affiliations:** 1 College of Medicine, King Saud Bin Abdulaziz University for Health Sciences, Riyadh, SAU; 2 College of Medicine, University of Jeddah, Jeddah, SAU; 3 College of Medicine, Princess Nourah Bint Abdulrahman University, Riyadh, SAU; 4 Medical Laboratory Technology, College of Applied Medical Sciences, Taibah University, Madinah, SAU; 5 College of Medicine, King Abdulaziz University, Rabigh, SAU; 6 College of Medicine, Taibah University, Madinah, SAU; 7 College of Medicine, University of Tabuk, Tabuk, SAU; 8 College of Medicine, King Khalid University, Abha, SAU; 9 College of Medicine, Umm Al-Qura University, Makkah, SAU; 10 College of Medicine, King Abdulaziz University, Jeddah, SAU; 11 College of Medicine, King Faisal University, Al-Hofuf, SAU; 12 Radiology, King Abdulaziz University Hospital, Jeddah, SAU

**Keywords:** capecitabine, chemotherapy efficacy, chemotherapy safety, colorectal cancer, s-1 chemotherapy

## Abstract

S-1 and capecitabine are both oral chemotherapy options often used to treat metastatic colorectal cancer (mCRC). Their effectiveness and side effects have generated clinical interest, particularly in deciding which one to use. This review aimed to directly compare the two regimens in terms of outcomes and tolerability.

Our study was conducted in accordance with PRISMA guidelines. A comprehensive search of PubMed/MEDLINE, Web of Science, and Google Scholar identified five eligible studies for the systematic review and meta-analysis. Primary outcomes were objective response rate (ORR) and disease control rate (DCR); secondary outcomes included progression-free survival (PFS) and overall survival (OS).

The analysis included 1,414 patients from five studies. S-1-based regimens showed a trend toward a higher ORR than capecitabine, with a pooled risk ratio of 1.14 (95% CI: 0.99-1.32; p = 0.07), though this difference did not reach statistical significance. There was no significant difference in DCR between the two groups (RR = 0.91, 95% CI: 0.46-1.83; p = 0.80), with substantial heterogeneity observed (I² = 66%). For the secondary outcomes, meta-analyses of PFS and OS could not be conducted due to insufficient data; however, descriptive analyses showed no statistically significant differences between the two regimens. Compared to capecitabine, S-1 was associated with significantly lower rates of hand-foot syndrome (HFS). However, the incidence of stomatitis was higher in the S-1 group.

S-1 was comparable in efficacy to capecitabine and may be particularly beneficial for patients who are intolerant to capecitabine-associated adverse events (AEs), especially HFS.

## Introduction and background

Colorectal cancer (CRC) is a leading cause of cancer-related deaths worldwide, ranking third globally for incidence and second for mortality, and is the third leading cause of cancer incidence and mortality in the U.S. [1,2]. Chemotherapy remains the primary first-line treatment for metastatic colorectal cancer (mCRC), with 5-fluorouracil (5-FU) administered either as monotherapy or in combination with oxaliplatin and/or irinotecan [3]. To improve convenience and avoid issues related to intravenous infusion, oral alternatives like capecitabine and S-1 were developed. S-1 is a fixed-dose combination of tegafur (a prodrug of 5-FU), gimeracil (which enhances 5-FU levels), and oteracil (which reduces gastrointestinal toxicity) [4]. Studies suggest that S-1 is effective against gastrointestinal cancers, including mCRC, and may be used alone or in combination with chemotherapy [5]. Similarly, capecitabine has demonstrated efficacy comparable to 5-FU, with improved tolerability and convenience [5,6]. However, capecitabine is associated with a high incidence of hand-foot syndrome (HFS), affecting up to 77% of patients, whereas S-1 has a lower risk of severe toxicity [6]. Despite their widespread use, direct comparisons between S-1 and capecitabine in mCRC are limited. This study aims to evaluate and compare their efficacy and adverse event (AE) profiles through a systematic review and meta-analysis, providing evidence to guide clinical decision-making.

## Review

Methodology

Literature Review

This systematic review was conducted according to the PRISMA 2020 statement, an updated guideline for reporting systematic reviews and meta-analyses [7]. The study protocol was registered in PROSPERO (ID: CRD42024571102) before data extraction. We systematically searched PubMed, Web of Science, and Google Scholar from inception to August 1, 2024. Rayyan software was used for screening and managing references (Qatar Computing Research Institute, Ar-Rayyan, Qatar). Rayyan is a tool developed by Ouzzani et al. to facilitate systematic reviews [8]. The search strategy included terms related to “S-1” AND “capecitabine” AND “colorectal cancer” AND “adverse event” AND “randomized controlled trials.” Only English-language articles meeting PICO (population, intervention, comparison, and outcome) criteria were included.

Study Selecting

English-language randomized controlled trials (RCTs) were conducted from 2012 to 2024. All articles met the following criteria: prospective phase II/III RCTs; patients histologically or cytologically diagnosed with metastatic colorectal carcinoma (mCRC, stage IV) as the population; intervention treatment with an S-1-based regimen; primary outcomes of objective response rate (ORR) and disease control rate (DCR); and secondary outcomes of progression-free survival (PFS), overall survival (OS), and AEs. Exclusion criteria included studies that did not directly compare S-1- and capecitabine-based regimens or lacked a control group. To reduce bias, non-randomized studies such as retrospective cohorts, prospective observational studies, and case-control studies were excluded. Single-arm phase II trials and early-phase studies without control groups were also excluded. Studies that did not report at least one predefined outcome - ORR, DCR, PFS, OS, or AEs - were ineligible. Ongoing clinical trials reported only as conference abstracts or trial listings without full-text, peer-reviewed publications were excluded. Non-original articles, including reviews, meta-analyses, editorials, letters, expert opinions, commentaries, and case reports, were also excluded. Only studies published in English and involving human subjects were considered. In cases of overlapping publications, the most recent and complete version was retained.

Process of screening and data extraction: Four independent reviewers screened titles and abstracts using the Rayyan web-based tool designed for systematic reviews. Studies that passed title and abstract screening underwent full-text review. Two investigators independently extracted data into a standardized Excel spreadsheet (Microsoft® Corp., Redmond, WA, USA). The following relevant data were extracted: first author, publication year, study type, region, sample size, patient demographics, ECOG (Eastern Cooperative Oncology Group) performance status, treatment regimens, ORR, DCR, PFS, OS, and AEs.

Assessment of Quality and Bias Risk

Version 2 of the Cochrane Collaboration’s tool for assessing risk of bias in randomized trials (RoB 2), developed by Higgins et al. [9], was used to assess risk of bias in the included studies. It is the recommended tool for assessing risk of bias in randomized trials. RoB 2 is structured into a fixed set of domains of bias, each focusing on different aspects of trial design, conduct, and reporting. Within each domain, a series of questions (signaling questions) aims to elicit information about trial features relevant to the risk of bias. Two reviewers independently assessed each study, with disagreements resolved through discussion or by involving a third reviewer. A proposed judgment about the risk of bias arising from each domain is generated based on answers to the signaling questions. Judgments can be ‘Low’, ‘High’, or ‘Some concerns’. As this study is a secondary analysis of previously published data, ethical approval was not required.

Statistical Analysis

The statistical analysis was conducted using Review Manager (RevMan; The Cochrane Collaboration, London, UK) for meta-analysis and Microsoft Excel for descriptive statistics. RevMan was used to calculate pooled risk ratios (RRs) with 95% confidence intervals (CIs) for dichotomous outcomes, including ORR, DCR, and AEs. Forest plots were generated, and statistical heterogeneity was assessed using the Chi-square test and I² statistic. A p-value < 0.05 was considered statistically significant. Microsoft Excel was used to compute frequencies and percentages for categorical variables, such as study design, country of origin, sex distribution, and ECOG performance status, and to summarize continuous variables - including age and sample size - using medians and ranges due to reporting limitations.

Results

Study Section and Characteristics

**Figure 1:**
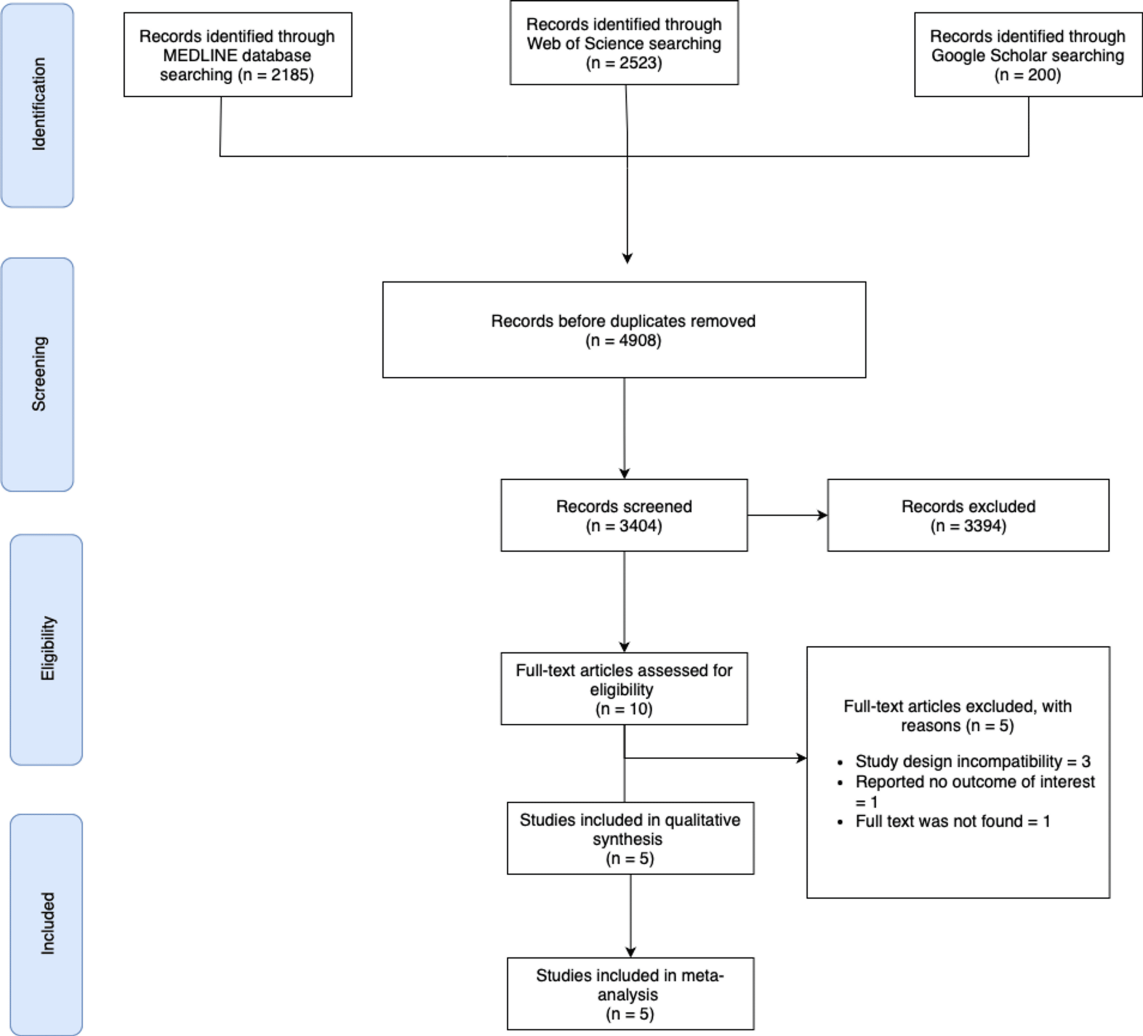
PRISMA flowchart illustrating the study selection process

The literature search identified 4,908 records: 2,185 from MEDLINE, 2,523 from Web of Science, and 200 from Google Scholar. After removing 810 duplicates, 4,098 records remained for screening. Two independent reviewers screened titles and abstracts, resulting in the exclusion of 3,394 records that did not meet the inclusion criteria. Ten full-text articles were assessed for eligibility. Of these, five were excluded: three due to study design incompatibility, one for not reporting relevant outcomes, and one due to the unavailability of the full text. Ultimately, five studies published between 2012 and 2018 were included in the qualitative synthesis [5,6,10-12]. The study selection process is summarized in the PRISMA flow diagram (Figure 1). All five studies included in the meta-analysis were RCTs. Among these, four were phase III trials with a noninferiority design, three of which were explicitly described as open-label. One study was a randomized phase II trial. Of the five studies included in this systematic review, three were conducted in South Korea, one in Japan, and one in the Netherlands, as summarized in Table 1.

**Table 1:** Basic demographics of the included studies ECOG, Eastern Cooperative Oncology Group

Study (Author, Year)	Country	Name of Journal Published	Study Design	Sample Size	Age Range (Years)	Male (%)	ECOG Performance Status
Hong et al. (2012) [5]	South Korea	The Lancet Oncology	Randomized, non-inferiority phase III trial	340	N/A	109 (32.1%)	0-2
Kwakman et al. (2017) [6]	Netherlands	Annals of Oncology	Randomized phase III trial	161	66-79	56 (34.8%)	0-2
Kim et al. (2014) [10]	South Korea	Journal of Cancer	Randomized phase II study	86	29-83	28 (32.6%)	0-2
Kim et al. (2015) [11]	South Korea	BMC Cancer	Randomized, non-inferiority phase III trial	340	N/A	109 (32.1%)	0-2
Yamada et al. (2018) [12]	Japan	Annals of Oncology	Randomized, open-label, phase III trial	487	22-87	143 (59.1%)	0-2

Patient Demographics and Treatment Details

The total number of patients across the included studies was 1,414 (sample size ranged from 86 to 487). The reported age range across the studies was 22 to 87 years, with an estimated mean age of 61.0 ± 10.0 years. Across all studies, a total of 970 patients were reported with a sex breakdown: of these, 512 (52.7%) were male and 458 (47.3%) were female. All included studies reported baseline ECOG performance status, with four studies including patients with scores of 0-2, and one study limiting inclusion to patients with scores of 0 or 1.

All studies compared S-1-based and capecitabine-based chemotherapy regimens, frequently in combination with oxaliplatin and/or bevacizumab. S-1 was administered orally at 30-40 mg/m² twice daily for 14 consecutive days, followed by a rest period of 7-14 days, depending on the study protocol. Capecitabine was typically administered at 1,000-1,250 mg/m² twice daily on days 1-14, with dose reductions for patients aged ≥70 years. Oxaliplatin was administered intravenously at 130 mg/m² on day 1 of each three-week cycle in most studies. In some regimens, bevacizumab was added at 5-7.5 mg/kg IV on day 1 or days 1 and 15. Treatment was continued until disease progression, unacceptable toxicity, or patient withdrawal, with maintenance therapy (S-1 or capecitabine) allowed after oxaliplatin discontinuation in selected protocols.

Risk of Bias Assessment

**Figure 2:**
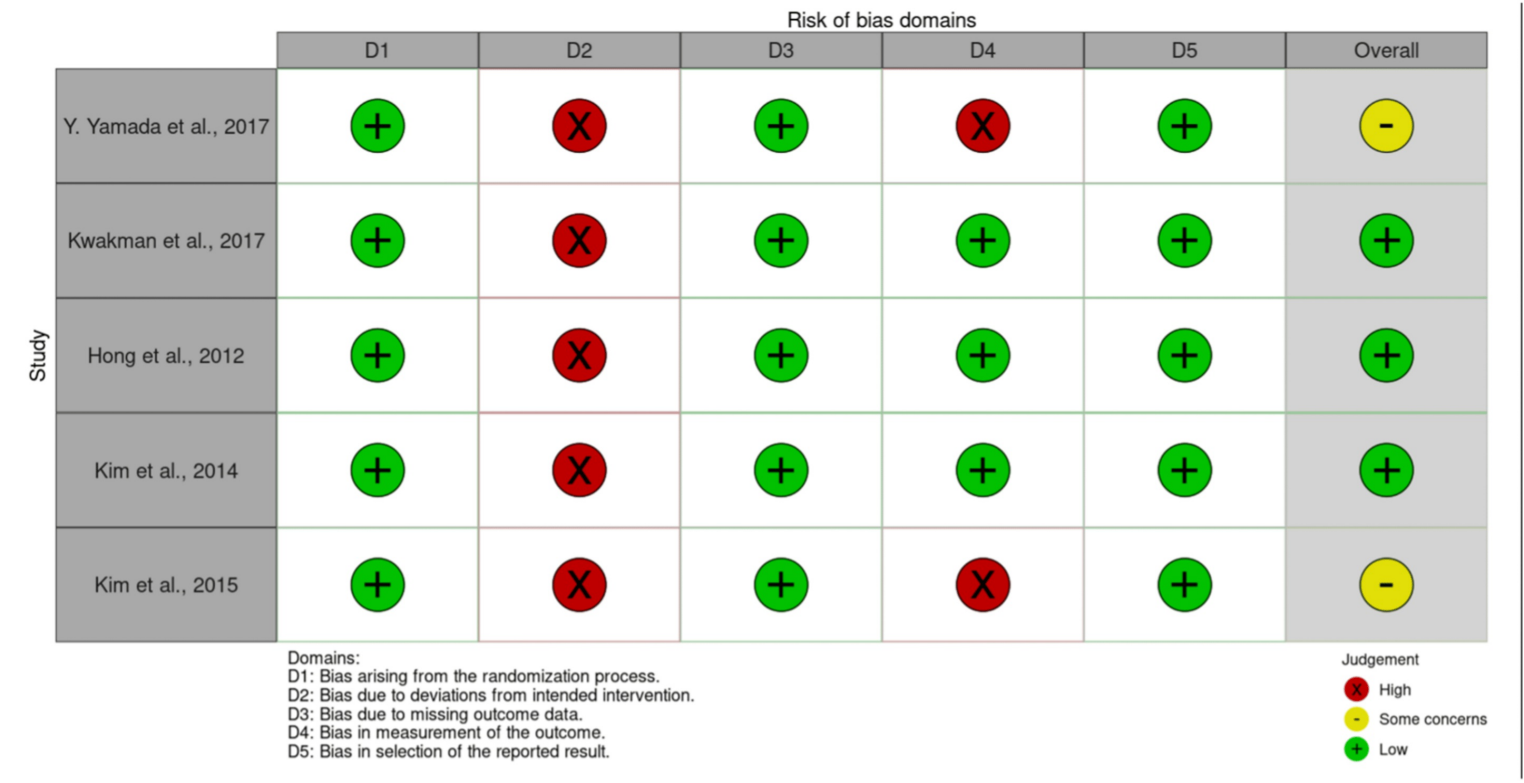
Risk of bias assessment summary for included studies using the Cochrane RoB 2.0 tool Green: low risk; Red: high risk; Yellow: some concerns Studies included: Hong et al. (2012) [5], Kwakman et al. (2017) [6], Kim et al. (2014) [10], Kim et al. (2015) [11], Yamada et al. (2018) [12]

The risk of bias in the five included RCTs was independently assessed by two reviewers using the Cochrane Risk of Bias 2.0 (RoB 2) tool, with specific judgments shown in Figure 2. The overall RoB 2 assessment indicated that three of the five studies were labeled as ‘low risk’ of bias, while two studies raised ‘some concerns’. Furthermore, a ‘high risk’ of bias was consistently identified across all five studies in Domain 2 (bias due to deviations from intended intervention), representing a key outcome of this assessment.

Objective Response Rate (ORR) 

**Figure 3:**
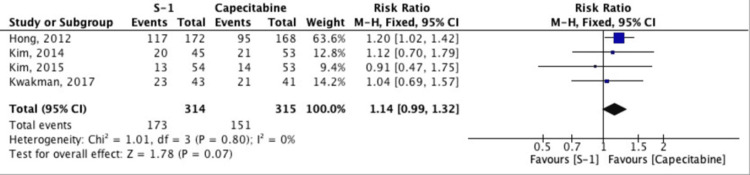
Forest plot comparing the objective response rate (ORR) between S-1 and capecitabine-based regimens Studies included: Hong et al. (2012) [5], Kwakman et al. (2017) [6], Kim et al. (2014) [10], Kim et al. (2015) [11]

A meta-analysis was conducted to compare ORR between patients treated with S-1 and those receiving capecitabine. Four studies were included, encompassing a total of 629 patients (S-1: n = 314; capecitabine: n = 315). The pooled RR for ORR was 1.14 (95% CI: 0.99-1.32; p = 0.07), favoring S-1, although the result did not reach statistical significance. The test for heterogeneity showed no significant heterogeneity across studies (I² = 0%, p = 0.80), indicating consistency among the included trials (Figure 3).

Disease Control Rate (DCR) 

**Figure 4:**
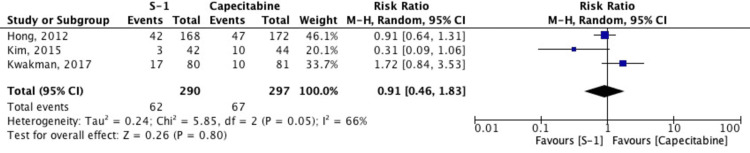
Forest plot comparing disease control rate (DCR) between S-1 and capecitabine-based regimens Studies included: Hong et al. (2012) [5], Kwakman et al. (2017) [6], Kim et al. (2015) [11]

DCR was reported in three studies and pooled using a random-effects model. The combined RR was 0.91 (95% CI: 0.46-1.83; p = 0.80), indicating no significant difference between the S-1 and capecitabine groups. Substantial heterogeneity was observed (I² = 66%), suggesting variability in study outcomes. Overall, the data do not support a clear superiority of either regimen in terms of DCR (Figure 4).

Progression-Free Survival (PFS)

Median PFS was reported in all five studies. In the largest study, the three-week and four-week S-1/irinotecan plus bevacizumab regimens achieved median PFS values of 13.2 months and 14.3 months, respectively, while the mFOLFOX6 and CapeOX arms showed 11.4 and 10.4 months, respectively [12]. Another trial reported a median PFS of 8.4 months in the S-1 group versus 8.2 months in the capecitabine group (HR 0.99, p = 0.93). In a third study, the SOX group had a longer median PFS than the CapeOX group in both the intention-to-treat (8.5 vs. 6.7 months) and per-protocol (8.5 vs. 6.6 months) populations [5]. A fourth study showed a median PFS of 7.1 months (SOX) vs. 6.3 months (CapeOX) (p = 0.10) [10]. The fifth study found a median PFS of 6.1 months (S-1) vs. 7.4 months (capecitabine) (p = 0.599) [11].

Overall Survival (OS)

Median OS was reported in four of the five studies [5,6,10,11]. The highest median OS was observed in the S-1/irinotecan plus bevacizumab group (34.9 months; 95% CI: 31.9-42.4), compared to 33.6 months (95% CI: 29.8-40.1) in the mFOLFOX6 or CapeOX group [12]. One study reported 12- and 18-month OS rates of 67% and 50% for capecitabine, and 62% and 41% for S-1 (HR 1.23; p = 0.32) [6]. In a third study, median OS was 21.2 months for SOX and 20.5 months for CapeOX in the intention-to-treat population [5]. Another trial showed similar OS: 19.0 vs. 18.5 months (HR 0.86; p = 0.19) [10]. 

Hematological Complications

**Figure 5:**
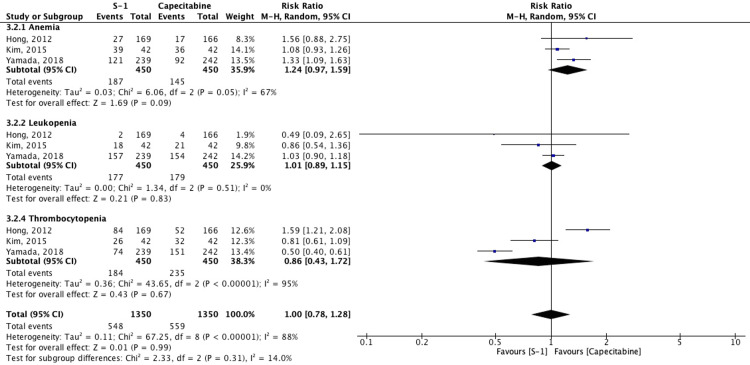
Forest plot comparing hematologic adverse events between S-1 and capecitabine regimens Studies included: Hong et al. (2012) [5], Kim et al. (2015) [11], Yamada et al. (2018) [12]

Three studies reported hematologic AEs, including anemia, leukopenia, and thrombocytopenia. Meta-analysis showed no statistically significant difference in overall hematologic toxicities between the S-1 and capecitabine groups (RR: 1.00, 95% CI: 0.78-1.28; I² = 88%). Subgroup analysis revealed a non-significant trend toward higher anemia risk with S-1 (RR: 1.24, 95% CI: 0.97-1.59), while thrombocytopenia appeared less frequent in S-1 arms, but with substantial heterogeneity (I² = 95%) (Figure 5).

Gastrointestinal Complications

**Figure 6:**
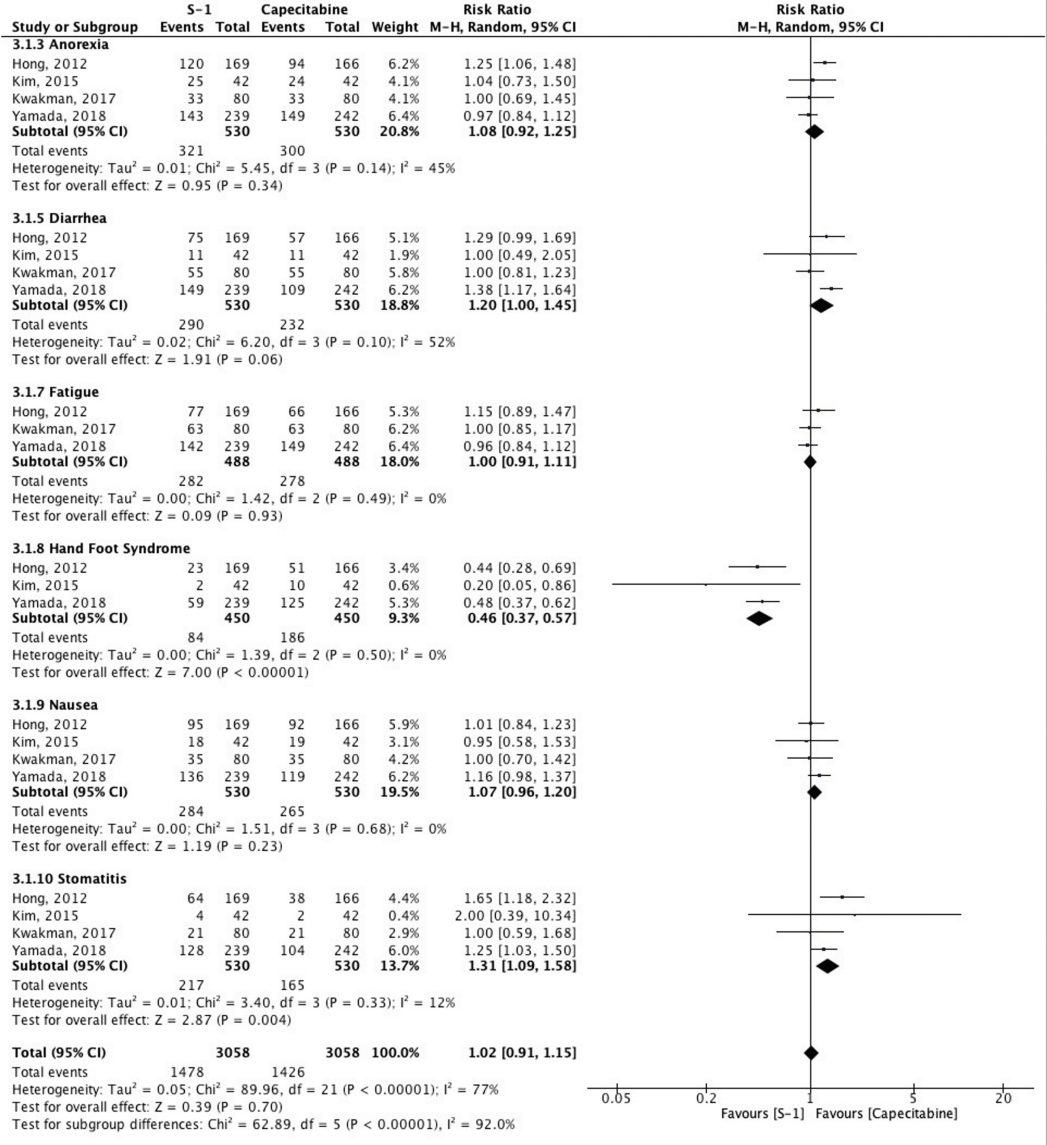
Forest plot comparing gastrointestinal adverse events between S-1 and capecitabine Studies included: Hong et al. (2012) [5], Kwakman et al. (2017) [6], Kim et al. (2015) [11], Yamada et al. (2018) [12]

Six GI-related AEs were included: anorexia, diarrhea, fatigue, HFS, nausea, and stomatitis. Notably, S-1 was significantly associated with lower rates of HFS (RR: 0.46, 95% CI: 0.37-0.57; I² = 0%) compared to capecitabine. Conversely, S-1 showed a significantly higher risk of stomatitis (RR: 1.31, 95% CI: 1.09-1.58; I² = 12%). Other complications showed no significant intergroup differences, although diarrhea and anorexia tended to be more frequent with S-1 (Figure 6).

Discussion

The choice between S-1 and capecitabine-based regimens remains uncertain. This systematic review and meta-analysis aimed to compare their efficacy and safety profiles to support treatment decision-making in this setting. We found that S-1-based regimens demonstrated a numerically higher ORR. In contrast, DCR showed no notable difference between the two groups, and findings were substantially heterogeneous across studies. Although not statistically significant, our pooled meta-analysis of four studies consistently favored the S-1 regimen in ORR, with no observed heterogeneity. This is consistent with the findings of Derksen et al., who reported that S-1 was as effective as capecitabine combined with 5-FU regimens, with a non-significant trend favoring S-1 (RR = 1.06; I² = 48%) [13]. This aligns with the findings of Chen et al., who reported a pooled ORR of 0.90 with moderate heterogeneity (I² = 46%, p = 0.10), similarly concluding no statistically significant difference between the regimens [14].

Regarding DCR, our pooled analysis showed no statistical difference between the two groups, with an RR of 0.91 (95% CI: 0.46-1.83; p = 0.80) and marked heterogeneity (I² = 66%). Neither our meta-analysis nor Chen et al. (pooled odds ratio: 0.92; 95% CI: 0.65-1.29; p = 0.61) showed a statistically significant difference between S-1 and capecitabine [14]. In contrast, Derksen et al. did not report DCR in their results [13].

To evaluate longer-term outcomes, we then assessed PFS, which is a crucial endpoint in mCRC studies. Although we could not perform a meta-analysis, PFS was reported in all five studies included in our review. Even though there was variability in median PFS values across the five studies - ranging from 13.2 to 14.3 months in the S-1/irinotecan plus bevacizumab arms - in the comparator arms, consisting of patients receiving mFOLFOX6 or CapeOX, median PFS ranged between 10.4 and 11.4 months, as reported in the largest included study. However, we found no consistent statistically significant difference between S-1-based and capecitabine-based regimens. The findings from Derksen et al. and Chen et al. were similar, with both meta-analyses concluding no statistically significant difference in PFS [13,14]. Derksen et al. reported a pooled hazard ratio (HR) for S-1-based therapy compared to 5-FU/capecitabine-based therapy of 0.95 (99% CI: 0.83-1.08), indicating non-inferiority [13]. Similarly, Chen et al. reported a pooled HR of 0.90 (95% CI: 0.75-1.08, p = 0.26) [14].

We then proceeded to analyze OS, another critical efficacy measure. OS was reported in four of the five studies included in our review. As with the PFS results, there was variability in the median OS across these four studies. For example, the largest study reported the highest median OS in the S-1/irinotecan plus bevacizumab group (34.9 months), compared to the mFOLFOX6 or CapeOX group (33.6 months). Nevertheless, the analysis revealed no significant difference in OS across treatment groups in any of the included studies. This lack of a significant difference in OS aligns with the findings of both Derksen et al. and Chen et al. [13,14]. Derksen et al. presented an overall HR for OS of 0.93 (99% CI: 0.81-1.07), indicating that S-1-based therapy is as effective as 5-FU/capecitabine-based therapy regarding OS [13]. Likewise, Chen et al. outlined a pooled HR for OS of 0.94 (95% CI: 0.78-1.13, p = 0.50), which also shows no statistically significant difference in OS between the treatment groups [14]. The absence of a statistically significant difference in OS was repeatedly noted across our included studies, as well as in the meta-analyses by Derksen et al. and Chen et al., confirming comparable long-term survival outcomes [13,14]. These results may help clinicians decide on treatment plans and affirm that the choice between these regimens can be determined by aspects beyond oncologic efficacy, considering the regimens’ AE profiles and patient values.

Regarding AEs, we found significantly lower rates of HFS with S-1-based regimens, which is consistent with the findings of both Derksen et al. and Chen et al. [13,14]. Additionally, S-1 was associated with higher rates of stomatitis, aligning with Derksen et al., whereas Chen et al. reported no significant difference in this outcome [13,14]. No consistent or statistically significant differences were observed between the regimens in terms of other hematologic or gastrointestinal AEs.

This study has several limitations. First, only five studies were included, which may limit the generalizability of the findings and reduce the statistical power to detect small differences. Second, a meta-analysis for our secondary outcomes of PFS and OS was not feasible due to inconsistent reporting and the absence of HRs in some studies, limiting the strength of our conclusions for these endpoints. Third, three of the included trials were open-label, which increases the risk of performance and detection bias. Lastly, variability existed in the chemotherapy backbones used across studies, with some regimens involving combinations with oxaliplatin, irinotecan, or bevacizumab. However, this heterogeneity was addressed by applying a random-effects model and formal heterogeneity analysis (I²). Future research with larger, high-quality RCTs is warranted to overcome these limitations and strengthen the evidence supporting these findings.

## Conclusions

This systematic review and meta-analysis found that S-1-based regimens are comparable in efficacy to capecitabine-based regimens for the treatment of mCRC. Additionally, S-1 was associated with a lower incidence of HFS. With additional research to support these findings, S-1 may serve as an effective alternative to capecitabine, particularly for patients who are unable to tolerate capecitabine or who are at higher risk for adverse events such as HFS.
